# Zukunft der Arbeit im Mittelstand: die Bedeutung der Arbeitsgestaltung für die Wandlungsfähigkeit von Unternehmen im Zeitalter der Digitalisierung

**DOI:** 10.1007/s41449-021-00290-7

**Published:** 2021-10-20

**Authors:** Christopher Brandl, Verena Nitsch

**Affiliations:** 1 Institut für Arbeitswissenschaft, RWTH Aachen University, Eilfschornsteinstr. 18, 52062 Aachen, Deutschland; 2 Fraunhofer-Institut für Kommunikation, Informationsverarbeitung und Ergonomie FKIE, Aachen, Deutschland

Seit Jahrzehnten verspricht die Digitalisierung Unternehmen Verbesserungen in der Bereitstellung und Nutzung von Informationen, effizientere Arbeitsprozesse, Produktivitätssteigerungen ebenso wie mehr Agilität und scheint damit unabdingbare Voraussetzung zu sein für das wirtschaftliche Wohl in einer globalen und zunehmend vernetzten Marktwirtschaft. Beschleunigung, Komplexität und Vernetzung sind dabei sowohl Konsequenz als auch Treiber der technikzentrierten Digitalisierung. Eine wichtige Aufgabe der arbeitswissenschaftlichen Forschung besteht in der Untersuchung der Auswirkungen des digitalen Wandels auf die menschliche Arbeit und der Ableitung von Maßnahmen für eine menschengerechtere Arbeitsgestaltung, welche oft auch digitale Lösungen beinhalten. Betrachtet man jedoch den digitalen Wandel in Deutschland genauer, so lässt sich vielerorts immer noch feststellen, dass dieser hauptsächlich in großen Unternehmen aktiv gestaltet wird. Dabei leistet die Arbeit in kleinen und mittleren Unternehmen zweifellos einen wichtigen Beitrag zur Wertschöpfung Deutschlands und sollte daher ebenso von den Möglichkeiten der Digitalisierung profitieren. Während vielen KMU die Notwendigkeit eines digitalen Wandels bereits grundlegend bewusst ist, so ist oft wenigen klar, dass er nicht nur neuer Technologien, sondern auch einer fachgerechten Arbeitsgestaltung bedarf.

Die Arbeitsgestaltung war bereits vor der Corona-Pandemie im Mittelstand angesichts oft knapper finanzieller Mittel und begrenzter Personalkapazitäten, gepaart mit hohem Zeit- und Konkurrenzdruck, herausfordernd (BMWi 2018). Die Digitalisierung erscheint vor diesem Hintergrund, dass bspw. ein Großteil der kleinen und mittleren Unternehmen bei der Steuerung ihrer Kernprozesse nach wie vor gedruckte Dokumente und veraltete Prozesse nutzt (BMWi 2016), ausbaufähig. Hier bedarf es nun passgenauer, anwendbarer sowie an den speziellen Herausforderungen des Mittelstands ausgerichteter Lösungen der Arbeitsgestaltung. Wenn auch eine Vielzahl der Führungskräfte im Mittelstand in der Digitalisierung eine Chance sieht, so erfolgen entsprechende Maßnahmen, wie bspw. die systematische Weiterqualifizierung von Beschäftigten, in der Praxis tatsächlich nur selten (BMWi 2016).

Im vorliegenden Themenheft werden Ergebnisse der Arbeitsforschung, insbesondere aus dem durch das Bundesministerium für Bildung und Forschung geförderten Forschungsschwerpunkt „Zukunft der Arbeit: Mittelstand – innovativ und sozial“ vorgestellt, die zuvor bestehende aber auch weitere Herausforderungen von KMU im digitalen Zeitalter aufgreifen. Einen Schwerpunkt stellt mit sechs Beiträgen das Themenfeld von Qualifikationen und Kompetenzen dar. Die Bedeutung dieses Themas für die Wandlungsfähigkeit von Unternehmen wird hierbei besonders deutlich: Wenn sich im Zuge der Transformation der Arbeit u. a. Arbeitsweisen und Arbeitsprozesse beschleunigt verändern, bedarf es zielgerichteter Anpassungen von Qualifikationen und Kompetenzen infolge erworbener Lernresultate der Arbeitsperson. Darüber hinaus zeichnet sich der Trend ab, das Lernen über einzelne Arbeitspersonen hinaus auch vermehrt im organisationalen Kontext zu betrachten.

Dem Themenfeld Qualifikationen und Kompetenzen widmen sich die ersten sechs Beiträge aus unterschiedlichen Perspektiven. Hegenberg und Schmidt untersuchen auf individueller Ebene Effekte eines Assistenzsystems basierend auf Augmented Reality für das Anlernen manueller und robotergestützter Montageprozesse. Am Beispiel von Montage- und Wartungstätigkeiten werden im Beitrag von Heinlein et al. niederschwellige Einsatzmöglichkeiten von Virtual Reality für die arbeitsintegrierte Komptenzentwicklung dargestellt und diskutiert. Ein Vorgehen für die prospektive und prozessbezogene Analyse zukünftiger Kompetenzen und Gestaltungen individueller und prozessorientierter Kompetenzentwicklungsmaßnahmen wird im Beitrag von Depenbusch et al. beschrieben. Die anschließende Studie von Tietz et al. weist auf die Wichtigkeit der sozialen Präsenz für einen erfolgreichen Wissensaustausch in virtuellen Teams hin. Die strategische Bedeutung des organisationalen Lernens bei der Bewältigung unternehmerischer Herausforderungen beschreiben Mynarek et al.; im Anschluß berichten Keller et al. über ein Vorgehen zur partizipativen Integration von Lern- und Assistenzsystemen im betrieblichen Alltag auf Basis eines organisationspädagogischen Ansatzes.

Eine erfolgreiche Digitalisierung setzt zudem auch die Akzeptanz spezifischer Maßnahmen voraus. Dieses Thema wird in zwei Beiträgen zur Akzeptanzforschung beleuchtet. Der Beitrag von Güsken et al. beschreibt, basierend auf empirischen Ergebnissen, förderliche und hinderliche Faktoren für die Unterstützung der Arbeit im Pflegebereich anhand einer Sensormatte. Die Studie von Sczogiel et al. zeigt auf, welche Parameter der Arbeitsgestaltung einen Effekt auf die Akzeptanz der Nutzenden bei der Einführung von Enterprise Collaboration Systemen haben können. Mögliche Zukunftsszenarien beim Einsatz von Smart Wearables für die Produktionsarbeit sowie deren Auswirkungen auf die Arbeitsgestaltung werden von Blumberg et al. erarbeitet.

Und schließlich erwies sich auch die COVID-19-Pandemie als Wandlungstreiber. Sie beschleunigte nicht nur die Digitalisierung in vielen Unternehmen, sondern verdeutlichte vielen auch die Bedeutung einer menschengerechteren Arbeitsgestaltung in Hinsicht auf die Produktivität und das Wohlbefinden von Arbeitskräften. Dies beleuchten Adam et al., die die Corona-Pandemie als Chance für Unternehmen sehen und zeitgleich untersuchen, welche Veränderungen im Kontext der Arbeit überregional und sektorenübergreifend auftreten, wie diese zu bewerten sind und welche neuen Arbeitsweisen sich auch in einer postpandemischen Zeit bewähren könnten.

Die gegenwärtige Bandbreite arbeitswissenschaftlicher Problem- und Fragestellungen im Mittelstand können durch die Beiträge in diesem Themenheft freilich nur angedeutet werden. Die damit verbundenen Herausforderungen als Chance unter Abwägung der Risiken zu betrachten und in Ansätzen integrierter Forschung Maßnahmen und Lösungen für eine humanorientierte und sozial nachhaltige Arbeitswelt zu erarbeiten und deren Auswirkungen wiederum zu evaluieren, ist ein vielversprechender Ansatz, den es weiter zu verfolgen gilt.

Wir danken den Autorinnen und Autoren für ihre positive Reaktion auf unsere Initiative und freuen uns, dem ZfA-Leserkreis ein Themenheft mit spannenden, zukunftsweisenden und praxisorientierten Beiträgen vorlegen zu können.
